# Biological Character of RetroNectin Activated Cytokine-Induced Killer Cells

**DOI:** 10.1155/2016/5706814

**Published:** 2016-06-28

**Authors:** Lu Han, Yi-Man Shang, Yong-Ping Song, Quan-Li Gao

**Affiliations:** ^1^Department of Immunology, Affiliated Cancer Hospital of Zhengzhou University and Henan Cancer Hospital, Zhengzhou, Henan 450008, China; ^2^Department of Hematology, Affiliated Cancer Hospital of Zhengzhou University and Henan Cancer Hospital, Zhengzhou, Henan 450008, China

## Abstract

Adoptive cell therapy (ACT) using autologous cytokine-induced killer (CIK) cells is a promising treatment for metastatic carcinomas. In this study, we investigated the impact of RetroNectin on the proliferation, phenotype alternation, cytokine secretion, and cytotoxic activity of CIK cells from pancreatic cancer patients. Furthermore, we treated 13 patients with metastatic or locally advanced pancreatic cancer using autologous RetroNectin-activated CIK cells (R-CIK cells) alone or in combination with chemotherapy. Compared with only CD3 activated CIK cells (OKT-CIK cells), R-CIK cells showed stronger and faster proliferative ability, with a lower ratio of spontaneous apoptosis. Moreover, this ability continued after IL-2 was withdrawn from the culture system. R-CIK cells could also secrete higher levels of IL-2 and lower levels of IL-4 and IL-5 versus OKT-CIK cells. There was no difference between OKT-CIK and R-CIK cells in cytotoxic ability against lymphoma cell line K562. In patients who received auto-R-CIK cell infusion therapy, the overall objective response rate was 23.1%. Median survival time (mOS) after first R-CIK cell infusion was 10.57 months; the 1-year survival rate was 38.5%. No serious toxicity was associated with R-CIK cell infusion. In conclusion, RetroNectin may enhance antitumor activity of CIK cells: it is safe for use in treating pancreatic cancer.

## 1. Introduction

Adoptive therapy using T cells for cancer therapy is a promising strategy that has curative potential and broad applicability. Cytokine-induced killer (CIK) cells are generated by in vitro expansion of peripheral blood lymphocytes (PBL) using anti-CD3 antibodies, IFN-*γ*, and IL-2 [1]. CIK cells are a heterogeneous population of effector T cells, some of which possessing non-MHC-restricted cytolytic activities against cancer cells [2]. Although clinical studies have confirmed the benefit and safety of CIK cell-based therapy for patients with hematopoietic and solid tumors, there are many factors impacting the clinical response due to the characteristics of ex vivo expanded lymphocyte cells. It was shown that the use of minimally cultured “young” less-differentiated tumor infiltrating lymphocytes (TILs), with stronger proliferative ability and higher levels of the costimulatory molecules CD27 and CD28, is an important factor for success [3]. RetroNectin is a chimeric peptide of recombinant* E. coli*-expressed human fibronectin fragments, with two functional domains which can interact with integrin molecules: very late antigen-4 (VLA-4, *α*4*β*1) and very late antigen-5 (VLA-5, *α*5*β*1) of the target cells. RetroNectin is always used to enhance retroviral mediated gene transduction by colocalizing target cells and virions on the RetroNectin molecules [4]. It was also found that T lymphocytes could be efficiently expanded by stimulation with a combination of immobilized RetroNectin and anti-CD3 mAb, resulting in a higher expansion fold and more naive T cells than other methods [5]. There were reports that RetroNectin activated lymphocytes could be used to treat solid tumors, proving that these cells were safe for clinical use [6, 7]. The detailed mechanism of how RetroNectin affects the biologic character of CIK cells, however, has not been systemically researched. In order to find an effective way to increase the number of high quality CIK cells for clinical use, we investigated the impact of using RetroNectin on proliferation, cytotoxic activity, phenotype alternation, and cytokine secretion of CIK cells. The CIK cells we used in this study were generated from the peripheral blood mononuclear cells of pancreatic cancer patients. We found that RetroNectin could increase the proliferative ability of the resultant CIK cells, increase growth time when IL-2 was withdrawn from the culture system, and make the cells secrete higher levels of IL-2 and lower levels of IL-4 and IL-5. Moreover, these CIK cells could be safely used to treat pancreatic cancer, as some of the patients get significant therapeutic efficacy.

## 2. Material and Methods

### 2.1. Culture of OKT-CIK and R-CIK Cells from Pancreatic Cancer Patients

50 mL of heparinized peripheral blood was obtained from five pancreatic cancer patients. All patients were approved by the Ethics Committee of Affiliated Cancer Hospital Zhengzhou University and signed an informed consent prior to initiation of lymphocyte cultures. Peripheral blood mononuclear cells (PBMCs) were separated from the blood by density gradient centrifugation. PBMCs were divided into two groups: group I and group II. Each group had 1 × 10^7^ cells. The PBMCs of group I were seeded into a 75 cm^2^ flask precoated with CD3 mAb (OKT3) (Cuba CIMAB SA, Cuba), while the PBMCs of group II were seeded into another 75 cm^2^ flask precoated with RetroNectin (Takara, Japan) and OKT3. Both groups of PBMCs were cultured with GT-T551 serum-free culture medium (Takara, Japan) supplemented with 2% autologous plasma, 1000 U/mL IFN-*γ* (Shanghai Kai Mao Biotechnology Co. Ltd., China), and 1000 U/mL IL-2 (Shandong Quangang Pharmaceutical Co. Ltd., China). After 4 days in culture, the two group cells in the 75 cm^2^ flasks were pipetted up completely to GT-T610 culture bags (Takara, Japan), with fresh medium containing 1000 U/mL IL-2 to 3 times the volume of the original medium added in the flask. Fresh culture medium containing 1000 U/mL IL-2 was added in the culture bags every 3 days. The cell product in the flask precoated with RetroNectin and OKT3 was named R-CIK cells, while the cell product in the flask precoated with OKT3 only was named OKT-CIK cells.

### 2.2. Culture of Leukemia Cell Line K562

K562 human immortalized myelogenous leukemia cells (ATCC) were cultured with RPMI-1640 medium (Gibico, USA) containing 10% fetal calf serum (Gibico, USA) at 37°C and 5% CO_2_ incubator. Fresh medium was changed every 3 days. The daily growth conditions of the cells were observed. Logarithmic growth phase of the K562 cells were used for cytotoxicity assays.

### 2.3. Checking Proliferative Activity of OKT-CIK and R-CIK Cells

After 4 days in culture, 5 mL medium containing OKT-CIK or R-CIK cells was extracted with a syringe from the 75 cm^2^ flasks and then cultured in a 25 cm^2^ flask in GT-T551 medium supplemented with 1000 U/mL of IL-2. The cell number was counted once every 3 days, and the expansion multiple was calculated by comparison with the original seeded cell number. Growth curve was drawn according to the cell expansion multiple.

We checked the continuing proliferative ability of the resultant OKT-CIK and R-CIK cells in the medium without IL-2 by performing IL-2 withdrawal tests. After 12 days in culture, parts of the OKT-CIK and R-CIK cells cultured in the culture bag were extracted and continued to be cultured in 24-well plates without IL-2, each sample in triplicate, with 1 × 10^4^ cells per well containing 1 mL medium. Cell numbers in the 24-well plate were counted every 2 days; the expansion multiple was calculated and the growth curve was drawn according to the multiple.

### 2.4. Measurement of Apoptosis

Apoptosis of the OKT-CIK and R-CIK cells was measured by Annexin V and Propidium Iodide (PI) staining using an Annexin V-FITC Apoptosis Detection kit (KeyGen, China). The cells were harvested and washed in cold PBS, then resuspended in 500 *μ*L binding buffer supplied by the kit, and stained with Annexin V-FITC 5 *μ*L, PI 5 *μ*L for 10 minutes at room temperature. The cells were analyzed by flow cytometry (BD Biosciences, San Jose, CA, USA) within 1 hour.

### 2.5. Phenotype of OKT-CIK and R-CIK Cells at Different Culture Times

OKT-CIK and R-CIK cells were measured on the 10th and 16th days after RetroNectin and OKT3 stimulating. The cells were harvested and washed in cold PBS and resuspended at 1 × 10^6^ in 100 *μ*L cold PBS consisting of 5% serum. The cells were stained for anti-CD3-APC, anti-CD4-PerCP, anti-CD8-PerCP, anti-CD27-FITC, anti-CD28-PE, anti-CD56-PE, and anti-PD-1-PE purchased from BD Biosciences (San Jose, CA, USA) for 20 minutes on ice. FACS analysis was performed by using a FACSCalibur and CellQuest software (BD Biosciences, San Jose, CA, USA).

### 2.6. CFSE-Based Cytotoxicity Assay

We harvested the logarithmic growth K562 cells by centrifuge as target cells. These cells were suspended at 1 × 10^6^ cells/mL and labeled with 1.25 *μ*mol/L CFSE (Invitrogen, USA) for 5 minutes at 37°C. The reaction was stopped by addition of an equal volume of RPMI1640 containing 10% fetal calf serum. Then, CFSE-labeled cells were centrifuged and resuspended in 10 mL RPMI1640 containing 10% fetal calf serum and incubated at 37°C for 30 minutes. Before use, CFSE-labeled K562 cells were washed two times with PBS and resuspended in GT-T551 medium at the cell concentration of 5 × 10^5^ cells/mL. Adjusting the concentration of OKT-CIK and R-CIK cells at 5 × 10^6^ cells/mL as effector cells, the effector and target cells were added at different effector-target ratios at 40 : 1 (400 *μ*L/100 *μ*L), 20/1 (330 *μ*L/165 *μ*L), and 10/1 (250 *μ*L/250 *μ*L) and were mixed to a final volume of 500 *μ*L in 48-well plates. The plates were then incubated in a humidified atmosphere of 5% CO_2_ and 37°C for 4 hours. To stain for dead cells, propidium iodide (final concentration 1 *μ*g/mL) was added 5 minutes before analysis. When using flow cytometry analysis, the CFSE^+^PI^−^ cells were alive target cells and the CFSE^+^PI^+^ cells were dead target cells. Spontaneous dead targets were obtained from targets incubated with medium alone and with PI staining which is positive. The percentage of CFSE^+^PI^+^ or PI^+^ K562 cells was performed by using a FACSCalibur and CellQuest software (BD Biosciences, San Jose, CA, USA). The percentage of specific target cell death (cytotoxicity) was then calculated as

(1) $$\begin{array}{c} \left(\;\frac{dead\;\;targets\;\;in\;\;the\;\;sample\;\;\left(\%\right)-spontaneously\;\;dead\;\;targets\;\;\left(\%\right)}{100\left(\%\right)-spontaneously\;\;dead\;\;targets\;\;\left(\%\right)}\;\right)\times 100\left(\;\%\;\right). \end{array}$$

### 2.7. Cytokine Secretion Assay

After 15 days in culture, OKT-CIK and R-CIK cells (1 × 10^5^) were plated at 1 × 10^5^ cells per well in a 96-well flat-bottom plate, with 1 × 10^5^ K562 tumor cells and 200 *μ*L GT-T551 medium without IL-2. After 24-hour coculture, supernatants were harvested and cytokine secretion was quantified by BD Cytometric Bead Array (CBA) Human Th1/Th2 Cytokine Kit II (BD Biosciences, San Jose, CA, USA) according to the protocol of the kit.

### 2.8. Patient Selection and Treatments

From September 2010 to December 2013, 13 patients with advanced pancreatic cancer from Affiliated Cancer Hospital of Zhengzhou University were treated by autologous R-CIK cells for at least 2 cycles, with or without chemotherapy according to the patients Eastern Cooperative Oncology Group performance status (ECOG PS) (Table 1). Eleven patients were diagnosed with metastatic pancreatic cancer; the other two were diagnosed with local advanced cancer. From 10 patients who were treated as first-line, three patients were treated after a failure with other treatments. The median age of these patients was 70 (49–79) years old, seven males and six females. Eight patients among them had histologically or cytologically confirmed pancreatic adenocarcinoma and the other five patients were diagnosed with computed tomography/magnetic resonance imaging (CT/MRI) combined with abnormal increase of cancer and tissue-specific marker carbohydrate antigen-199 (CA-199). Patients whose expected survival time was less than 2 months were excluded from the trial.

The protocol of R-CIK cells culture from the patients was similar to the protocol described above. Briefly, 50 mL of heparinized peripheral blood was obtained from the patients, after 11~16 days in culture, and the R-CIK cells were harvested and infused to those patients who were fit to clinical use through quality control. All patients who were entered into the clinical protocols signed informed consent forms that were approved by the Ethics Committee of Affiliated Cancer Hospital of Zhengzhou University prior to initiation of lymphocyte cultures.

Six patients received S-1 (Jiangsu Hengrui Medicine Co. Ltd., China) based chemoimmunotherapy; the dose of S-1 used in each cycle was 80, 100, or 120 mg/d according to body-surface area on days 1 through 14 of a 21-day cycle. After 24~48 hours of the last day's S-1 oral administration, 3~5 × 10^9^ autologous R-CIK cells were intravenous drop infused to each patient, followed by 2 million units of interleukin-2 intravenous drop infusion each day, for 3 days. Three patients received gemcitabine (Haosenh Pharmaceutical Co., China) based chemoimmunotherapy; the dose of gemcitabine used in each cycle was 1000 mg/m^2^ on days 1 and 8 of a 21-day cycle. After 24~72 hours of the second gemcitabine dose infusion, 3~5 × 10^9^ autologous R-CIK cells were intravenous drop infused to each patient, followed by 2 million units of interleukin-2 intravenous drop infusion each day, for 3 days. These patients received 1~6 cycles chemoimmunotherapy altogether, according to the patients Eastern Cooperative Oncology Group performance status (ECOG PS). After those chemoimmunotherapy cycles were completed, 1~4 doses of more R-CIK cells were infused after the last chemotherapy cycle; each dose contained about 3~5 × 10^9^ R-CIK cells. Four patients received only R-CIK cells infusion without treatment of chemotherapy due to age or refusal to use chemotherapy drugs. These patients received 2~10 doses of R-CIK cells infusion, respectively, twice a month; each dose contained about 3~5 × 10^9^ R-CIK cells. After R-CIK cells infusion, patients received 2 million units of interleukin-2 intravenous drop infusion each day, for 5 days.

### 2.9. Assessment of Response and Toxicity

The staging of pancreatic cancer was diagnosed according to UICC/AJCC 2002 criteria. Tumor response was determined according to the National Cancer Institute (Bethesda, MD) Response Evaluation Criteria in Solid Tumors (RECIST1.1) [8]. Patients were assessed serially using CT/MRI of chest, abdomen, pelvis, and brain and technetium bone scan. Tumor evaluation was done 2 months after treatment start. Overall survival was calculated from the date of therapy initiation until the date of death or December 31, 2013. Adverse events were evaluated according to World Health Organization (WHO) criteria.

### 2.10. Statistical Analysis

Overall survival (OS) was calculated from initiation of therapy to death, and patients alive were censored at the time of last contact. We retrospectively analyzed tumor response and overall survival of 13 patients. Distributions of survival time and rate were estimated by using the Kaplan-Meier method. Median survival time along with 95% confidence intervals (CI) was reported. All calculations were conducted using the SPSS 17.0 software.

## 3. Results

### 3.1. RetroNectin Activated CIK Cells Had Stronger Proliferative Ability

**Figure 1:**
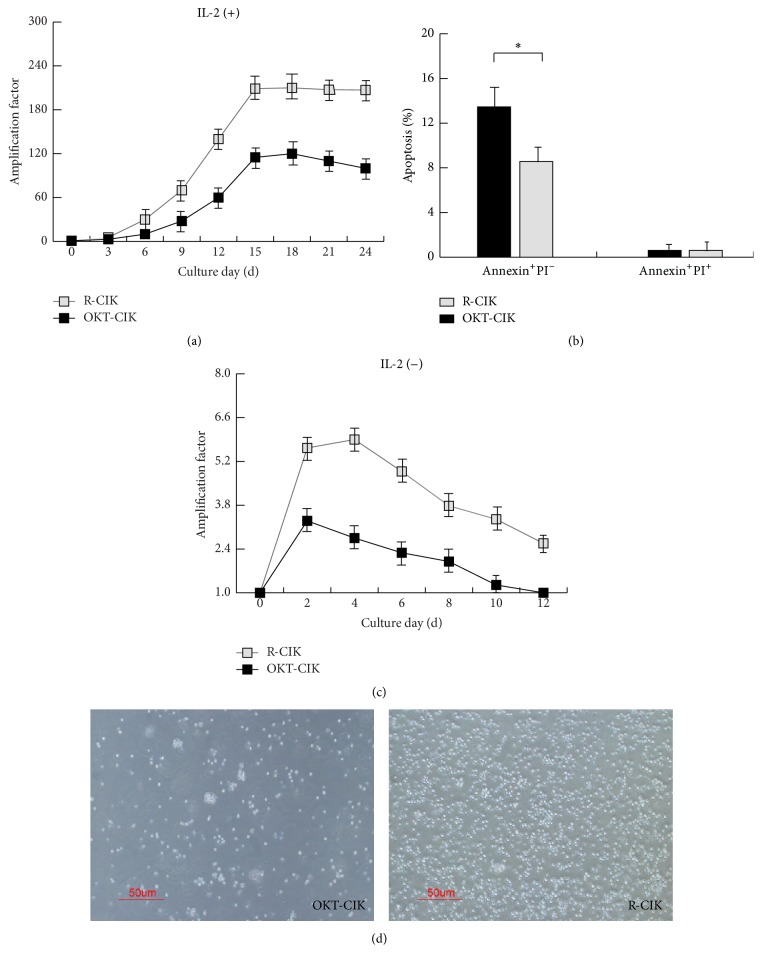
RetroNectin activated CIK cells had stronger proliferative ability. (a) Proliferative curve of OKT-CIK and R-CIK cells. The proliferative speed of R-CIK cells was much higher than that of OKT-CIK cells, *n* = 5. (b) Mean percentage of OKT-CIK and R-CIK cells undergoing early apoptosis (Annexin^+^PI^−^) and late apoptosis/necrosis (Annexin^+^PI^+^). ^**∗**^ *P* < 0.05 for the comparison, *n* = 5. (c) Continual proliferative curve of OKT-CIK and R-CIK cells in medium without IL-2. R-CIK cells could continue expanding 4 days after IL-2 was withdrawn from the medium, and the maximum average amplification is 6 times. OKT-CIK cells could only continue expanding 2 days in the same condition, and the maximum average amplification is 3 times, *n* = 5. (d) Shape of cultured OKT-CIK and R-CIK cells (400x).

Three days after activation by OKT3 alone or OKT3 combined with RetroNectin, both OKT-CIK and R-CIK cells began a logarithmic growth stage, but the growth speed of R-CIK cells was much higher than that of OKT-CIK cells (Figure 1(a)). Both OKT-CIK and R-CIK cells achieved growth platform after 15 days in culture: the OKT-CIK cells displayed 100 times amplification at this time, while the R-CIK cells displayed 200 times amplification (Figure 1(a)). Coincident with the growth speed, the spontaneous apoptosis of R-CIK cells was lower than that of OKT-CIK cells (Figure 1(b)). After IL-2 was withdrawn from the medium, R-CIK cells could continue growing to 6 times amplification, while OKT-CIK cells could only grow to 3 times amplification (Figure 1(c)). These results indicate that R-CIK cells activated by RetroNectin have much stronger proliferative ability than only CD3 activated CIK cells. There were also some shape differences between OKT-CIK and R-CIK cells; OKT-CIK cells displayed easily formed cells aggregates, while R-CIK cells displayed dispersed growth (Figure 1(d)).

### 3.2. Subpopulation Cells in OKT-CIK and R-CIK Cells Changed at Different Culture Times

**Figure 2:**
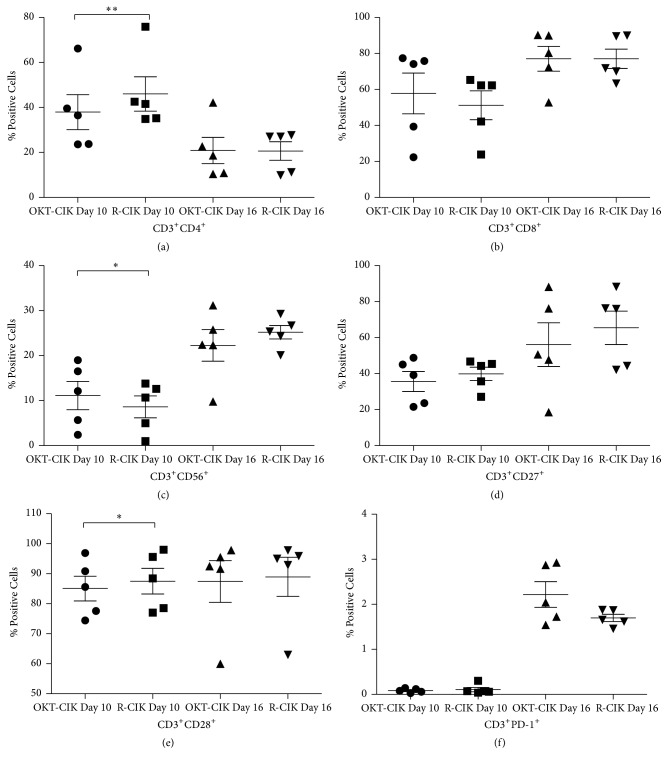
Composition of T subpopulation cells at different culture times. (a) CD3^+^CD4^+^ T cells cultured on the 10th and 16th day. (b) CD3^+^CD8^+^ T cells cultured on the 10th and 16th day. (c) CD3^+^CD56^+^ T cells cultured on the 10th and 16th day. (d) CD3^+^CD27^+^ T cells cultured on the 10th and 16th day. (e) CD3^+^CD28^+^ T cells cultured on the 10th and 16th day. (f) CD3^+^PD-1^+^ T cells cultured on the 10th and 16th day. CD3^+^CD4^+^ and CD3^+^CD28^+^ cells were higher in R-CIK cells, while CD3^+^CD56^+^ cells were lower in R-CIK cells on the 10th day, but they became equal on the 16th day. There was no difference seen between the OKT-CIK and R-CIK cells when compared to other subpopulation cells. As the culture time prolonged, CD3^+^CD8^+^ cell counts were raised in both OKT-CIK and R-CIK cells, and CD3^+^CD4^+^ cells decreased in both OKT-CIK and R-CIK cells at the same time. The % of the cells shows the % of cells in all cell population in the culture. ^**∗**^ *P* < 0.05; ^**∗∗**^ *P* < 0.01 for the comparison, *n* = 5.

We analyzed the cell subpopulations in OKT-CIK and R-CIK cells cultured on the 10th and 16th days, including CD3^+^CD4^+^, CD3^+^CD8^+^, CD3^+^CD56^+^, CD3^+^CD27^+^, CD3^+^CD28^+^, and CD3^+^PD-1^+^ cells. As shown in Table 2 and Figure 2, the percentage of CD3^+^CD4^+^ cells and the percentage of CD3^+^CD28^+^ cells were higher in R-CIK cells on the 10th day (*P* < 0.05), but they became equal on the 16th day. Conversely, the percentage of CD3^+^CD56^+^ cells was lower in R-CIK cells on the 10th day (*P* < 0.05); it also became equal on the 16th day. There was no difference seen between the OKT-CIK and R-CIK cells when compared to other subpopulation cells (*P* > 0.05).

As the culture time prolonged, CD3^+^CD8^+^ cell counts were raised in both OKT-CIK and R-CIK cells; their percentages were higher on the 16th day than on the 10th day. Correspondingly, CD3^+^CD4^+^ cells decreased in both OKT-CIK and R-CIK cells at the same time.

### 3.3. Cytokines Secreted by OKT-CIK and R-CIK Cells

**Figure 3:**
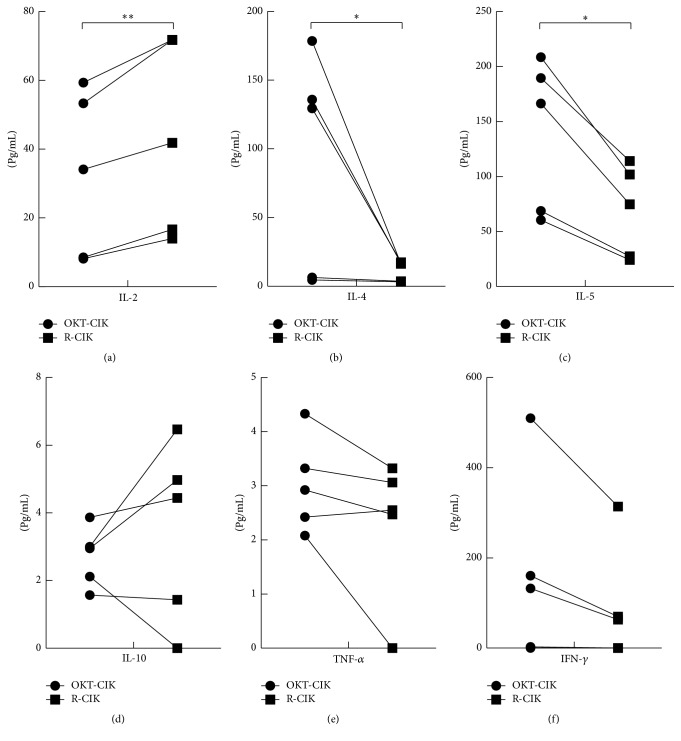
Cytokine secreted by OKT-CIK and R-CIK cells. (a) IL-2 was secreted by OKT-CIK and R-CIK cells. (b) IL-4 was secreted by OKT-CIK and R-CIK cells. (c) IL-5 was secreted by OKT-CIK and R-CIK cells. (d) IL-10 was secreted by OKT-CIK and R-CIK cells. (e) TNF-*α* was secreted by OKT-CIK and R-CIK cells. (f) IFN-*γ* was secreted by OKT-CIK and R-CIK cells. R-CIK cells secreted higher IL-2, while they secreted lower level of IL-4 and IL-5. There was no difference between the OKT-CIK and R-CIK cells when compared to IL-10, TNF-*α*, and IFN-*γ* (*P* > 0.05). ^**∗**^ *P* < 0.05; ^**∗∗**^ *P* < 0.01 for the comparison, *n* = 5.

We checked the cytokines secreted by OKT-CIK and R-CIK cells when they were cocultured with K562 cells using the Cytometric Bead Array (CBA) Human Th1/Th2 Cytokine Kit. The cytokines checked included IL-2, IL-4, IL-5, IL-10, TNF-*α*, and IFN-*γ*. As shown in Figure 3, compared with OKT-CIK cells, R-CIK cells secreted higher IL-2, while they secreted lower level IL-4 and IL-5 (*P* < 0.05). There was no difference between the OKT-CIK and R-CIK cells when compared to IL-10, TNF-*α*, and IFN-*γ* (*P* > 0.05). These results indicate that RetroNectin can promote the activity of Th1 cells.

### 3.4. Cytotoxicity of OKT-CIK and R-CIK Cells to K562 Cells

**Figure 4:**
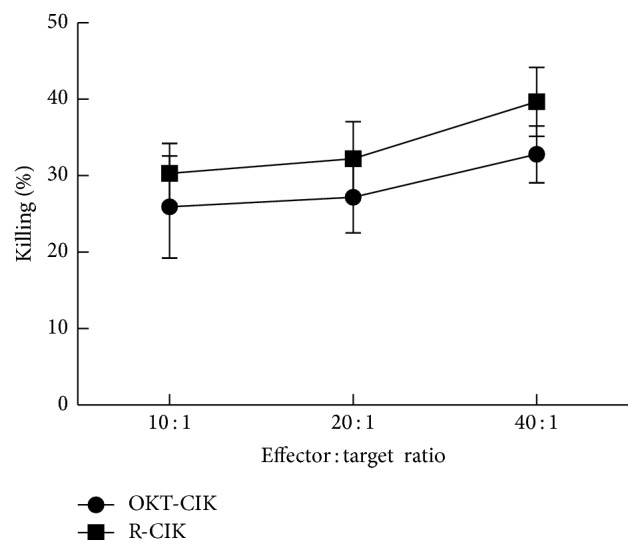
Cytotoxicity of OKT-CIK and R-CIK cells against K562 cells. Both OKT-CIK and R-CIK cells could kill K562 cells at different effect or target ratio, but there was no statistical difference between the two groups. *P* > 0.05, *n* = 5.

The OKT-CIK and R-CIK cells were tested for cytotoxicity against the K562 tumor cells, measured by CFSE/PI double staining and flow cytometry analysis. As shown in Figure 4, both OKT-CIK and R-CIK cells could kill K562 cells at different effector/target ratio, but there was no statistical difference between the two group cells (*P* > 0.05).

### 3.5. Tumor Response and Prognosis of R-CIK Cell Treated Pancreatic Cancer Patients

**Figure 5:**
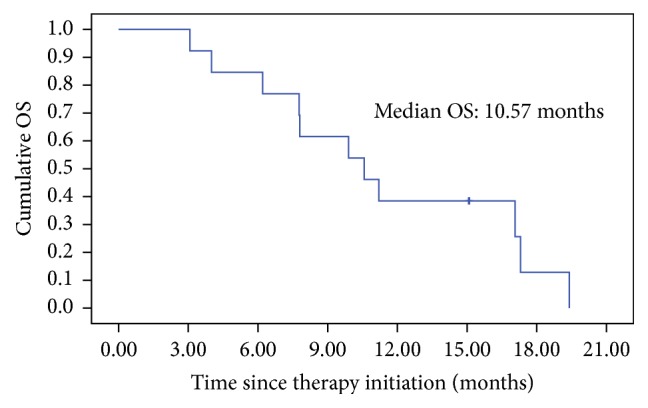
Overall survival measured using the Kaplan-Meier method. The median survival time after first R-CIK cells infusion was 10.57 months. One-year survival rate was 38.5%.

Of the 13 pancreatic cancer patients studied, one patient (7.7%) achieved a complete remission (CR): this patient had local relapsed pancreatic adenocarcinoma after surgery and preventive radiotherapy. Two patients (15.4%) who received chemotherapy combined R-CIK cells infusion achieved a partial remission (PR). Eight patients (61.5%) had a stable disease (SD). The overall objective response rate (CR+PR) was 23.1% and the disease control rate (CR+PR+SD) was 84.6%. The median survival time (mOS) after first R-CIK cells infusion was 10.57 months (95% CI, 6.6 to 14.6 months; Figure 5). The 1-year survival rate was 38.5%.

### 3.6. Treatment Toxicity

The distributions of side effects were shown in Table 1. No toxic death was observed. Common toxicities consisting of bone marrow suppression, anorexia, and fatigue were mainly in the chemotherapy group. One patient who received solo R-CIK infusion had toxicity of mild fever. The toxicities in the chemotherapy group were more frequent and serious than those in the group without chemotherapy.

## 4. Discussion

CIK cells are generated by culturing peripheral blood lymphocytes (PBL) with interferon-*γ* (INF-*γ*), monoclonal antibody against CD3 (anti-CD3), and IL-2 in a particular time schedule. The cytokines INF-*γ* and IL-2 are crucial for the cytotoxicity of the cells and anti-CD3 provides mitogenic signals to T cells for proliferation [9]. CD3^+^CD8^+^CD56^+^ cells are referred to as natural killer T (NK-T) cells and represent the cell type with the greatest cytotoxicity in the CIK cell population. Among different adoptive lymphocyte therapies, CIK cells have a particularly advantageous profile as these cells are easily available, have a high proliferative rate, own non-MHC-restricted cytotoxicity ability, and exhibit a high antitumor activity [10]. There are reports that CIK cells could even kill cancer stem cells [11, 12]. Both autologous and allogeneic CIK cells have been used in phases 1 and 2 of clinical trials for the treatment of different tumor types. In these trials, they displayed limited toxicity, whereas evidence has been obtained that they exert antitumor activity [13]. Although clinical studies have confirmed benefit and safety of CIK cell-based therapy for patients with malignancies, the efficacy was usually low, so enhancement of the potency of CIK cells is very important. In order to find an easy way to improve the efficacy of CIK cells for clinical use, we added RetroNectin to the traditional CIK cell culture system. We found that, after using RetroNectin, most of the biologic characters of the R-CIK cells were similar to unaltered CIK cells. For example, CIK and R-CIK cells had similar T cell subpopulations after 16 days in culture; their cytotoxicity abilities against lymphoma cell line K562 cells were also equal. RetroNectin, however, could make R-CIK cells secrete more Th1 cell-associated cytokine IL-2 and secrete less Th2 cell-associated cytokine IL-4 and IL-5, indicating that RetroNectin could enhance the activity of Th1 cells, while suppressing the activity of Th2 cells. This is a clinically significant advantage for R-CIK cells, since cancer immunotherapy needs the help of Th1 cells, while the Th2 cells always inhibit antitumor activity of immune system [14]. Another advantage of RetroNectin is that it could promote the proliferative ability of CIK cells; the R-CIK cells' growth speed was faster, with a lower ratio of spontaneous apoptosis. This characteristic of RetroNectin could shorten the culturing time in vitro to get a certain amount of cells for clinical use; it could also improve the quality of resultant lymphocytes, as it has been reported that the use of less-differentiated TILs with stronger proliferative ability was an important factor for success [3]. More importantly, after IL-2 was withdrawn from the medium, R-CIK cells continue growing for a longer time and expanded double numbers of OKT-CIK cells which were not excited by RetroNectin. This characteristic of R-CIK cells made them have two merits for clinical use. First, traditional CIK cells should stop growing soon after they were infused into the patient blood, because the concentration of IL-2 in the patient's body is very low, while RetroNectin activated R-CIK cells may continue to grow for a longer time. Second, the patient's blood may be the most favorable culture medium for R-CIK cells' growth: if the patient is given IL-2 several days after infusion, the cells may continue to grow for more days in the patient body, so the therapeutic number of R-CIK cells needed for cancer patients could be less than that of traditional CIK cells. Indeed, we used average 4 × 10^9^ R-CIK cells in each dose to treat pancreatic cancer in our study; it seems that R-CIK cells could work at this order of magnitude, while other researchers usually use more than 1 × 10^10^ CIK cells to treat cancer patients [15, 16].

Pancreatic cancer is a relatively common malignancy, with around 43,000 new cases diagnosed in the U.S. in 2012. The majority of patients have advanced pancreatic cancer at the time of diagnosis. Advanced disease is associated with a dismal outcome, with a median survival of 3–6 months [17]. Although advanced pancreatic cancer shows a modest response to chemotherapy, different studies suggest that pancreatic cancer can elicit antitumor immune responses [18–21]; thus, immunotherapy could be of great importance in treatment. Recently, immunotherapy has been shown to be a novel approach with the potential to function alone or in concert with traditional therapies: results in many clinical trials have shown improved survival without added toxicity. In a large retrospective cohort analysis, 138 palliative treatment pancreatic cancer patients had added treatment with dendritic cells. The therapy was well tolerated and no serious side effects were observed. Median survival time was 8.9 months. Median survival was significantly higher in the group of patients who started immunotherapy within 2 months following diagnosis or repeated immunotherapy [22]. CIK cells have been used to treat many types of malignant tumors in clinical trials, where they displayed limited toxicity and some efficacy. And, in our previous study, we found that R-CIK cells combined with conventional therapies could improve the prognosis of metastatic brain tumor patients, especially of those with adenocarcinoma of the lung [23]. However, there were no clinical trial reports on CIK treatment in pancreatic cancer. In the present study, we use R-CIK cells to treat 13 pancreatic cancer patients alone or combined with chemotherapy. Eleven patients were diagnosed with metastatic pancreatic cancer; the other two were local advanced. The overall objective response rate was 23.1%, including one patient who achieved a complete remission (CR) after receiving R-CIK cells infusion alone; two patients achieved a partial remission (PR) after receiving chemotherapy combined with R-CIK cells infusion. Eight patients (61.5%) had a stable disease (SD). The overall objective response rate (CR+PR) was 23.1% and disease control rate (CR+PR+SD) was 84.6%. The median survival time (mOS) after first R-CIK cell infusion was 10.57 months, and the 1-year survival rate was 38.5%. There was no serious toxicity associated with R-CIK cells infusion; one patient had a mild fever. Although the patients' number was low, it seems that R-CIK greatly improved the survival time of the metastatic pancreatic cancer.

In conclusion, our results showed that after activation by RetroNectin, CIK cells displayed stronger and faster proliferative ability, even in an environment without extraneous IL-2, and enhanced the activity of Th1 cells. The R-CIK cells can be safely used to treat pancreatic cancer patients, and some patients can experience significant results from the treatment. Due to the limited number of cases in our study, however, additional studies should be performed.

## Figures and Tables

**Table 1:** The baseline data of 13 pancreatic cancer patients.

Code number	Sex/age (years)^#^	Diagnosis	Chemotherapy	CIK cycles	Treatment response	OS (months)	Adverse events
1	F/61	Hepatic metastasis of pancreatic adenocarcinoma after operation	S-1	2	PD	3.07	II° Diarrhea
2	M/58	Hepatic metastasis of pancreatic cancer	Gemcitabine	11	PD	4.00	III° Thrombocytopenia
3	F/76	Hepatic metastasis of pancreatic cancer	No	6	SD	6.20	Mild fever
4	M/49	Hepatic metastasis of pancreatic cancer	S-1	2	SD	7.77	/
5	F/49	Liver and lymph nodes metastasis of pancreatic adenocarcinoma after operation	S-1	12	SD	7.80	Fatigue and anorexia
6	M/70	Lung metastasis of pancreatic head cancer	Gemcitabine	6	SD	9.90	III° Diarrhea and II° bone marrow suppression
7	M/71	Liver and adrenal metastasis of pancreatic cancer	S-1	4	SD	10.57	/
8	M/58	Abdominal metastasis of pancreatic adenocarcinoma	Gemcitabine	9	SD	11.2	II° Bone marrow suppression
9	F/79	Bone and liver metastasis of pancreatic adenocarcinoma after operation	No	4	SD	17.07	/
10	M/72	Hepatic metastasis of pancreatic adenocarcinoma	S-1	20	PR	17.30	/
11^*∗*^	M/52	Gastric and duodenal metastasis of pancreatic head adenocarcinoma	No	12	SD	19.07	/
12	F/74	Lung metastasis of pancreatic head adenocarcinoma	S-1	25	PR	19.40	Fatigue and anorexia
13^*∗*^	F/77	Peritoneal metastasis of pancreatic adenocarcinoma after operation	No	40	CR	36.53	/

^#^M, male; F, female.

^*∗*^Patient alive.

CR: complete remission, PR: partial remission, SD: stable disease, and PD: progressive disease.

**Table 2:** The cell phenotype of CIK at different culture time ($\overset{-}{x}\pm s$, *n* = 5).

Phenotype	Day 10 (%)		Day 16 (%)	
	OKT-CIK	R-CIK	OKT-CIK	R-CIK
CD3^+^CD4^+^	37.95 ± 17.38^*∗∗*^	46.01 ± 17.02^*∗∗*^	20.90 ± 12.94	20.66 ± 9.16
CD3^+^CD8^+^	57.82 ± 25.32	51.20 ± 17.83	77.00 ± 12.11	77.04 ± 15.49
CD3^+^CD56^+^	11.14 ± 7.02^*∗*^	8.66 ± 5.36^*∗*^	22.21 ± 7.85	25.18 ± 3.38
CD3^+^CD27^+^	35.62 ± 12.43	39.84 ± 8.33	56.11 ± 27.11	65.42 ± 20.82
CD3^+^CD28^+^	85.06 ± 9.25^*∗*^	87.49 ± 9.58^*∗*^	87.41 ± 15.57	88.93 ± 14.60
CD3^+^PD-1^+^	0.09 ± 0.21	0.12 ± 0.12	2.22 ± 0.64	1.70 ± 0.18

^*∗*^ *P* < 0.05; ^*∗∗*^ *P* < 0.01 for the comparison.
